# Performance of an RNA-Based Next-Generation Sequencing Assay for Combined Detection of Clinically Actionable Fusions and Hotspot Mutations in NSCLC

**DOI:** 10.1016/j.jtocrr.2022.100276

**Published:** 2022-01-10

**Authors:** Patrice Desmeules, Dominique K. Boudreau, Nathalie Bastien, Marie-Chloé Boulanger, Yohan Bossé, Philippe Joubert, Christian Couture

**Affiliations:** a Service of Anatomic Pathology and Cytology, Institut Universitaire de Cardiologie et de Pneumologie de Québec—Université Laval, Québec City, Québec, Canada; b Research Center, Institut Universitaire de Cardiologie et de Pneumologie de Québec—Université Laval, Québec City, Québec, Canada

**Keywords:** Actionable driver mutations, Anchored multiplex PCR, Next-generation sequencing, Non–small cell lung cancer

## Abstract

**Introduction:**

With its expanding list of approved and emerging therapeutic indications, NSCLC is the exemplar tumor type requiring upfront assessment of several biomarkers to guide clinical management. Next-generation sequencing allows identification of different types of molecular alterations, each with specific analytical challenges. Library preparation using parallel DNA and RNA workflows can overcome most of them, but it increases complexity of laboratory operations, turnaround time, and costs. We describe the performance characteristics of a 15-gene RNA panel on the basis of anchored multiplex polymerase chain reaction for combined detection of clinically relevant oncogenic fusion transcripts and hotspot small variants.

**Methods:**

Formalin-fixed, paraffin-embedded NSCLC clinical samples (N = 58) were used along cell lines and commercial controls to validate the assay’s analytical performance, followed by an exploratory prospective cohort (N = 87).

**Results:**

The raw assay sensitivity for hotspot mutations and fusions was 83% and 93%, respectively, reaching 100% after filtering for key assay metrics. Those include quantity and quality of input of nucleic acid and sequencing metric from primers on housekeeping genes included in the assay. In the prospective cohort, driver alterations were identified in most cases (≥58%).

**Conclusions:**

This ultrafocused RNA–next-generation sequencing assay offers an advantageous option with single unified workflow for simultaneous detection of clinically relevant hotspot mutations and fusions in NSCLC, focusing on actionable gene targets.

## Introduction

Lung cancer has the highest cancer-related mortality rate among solid tumors, and NSCLC is diagnosed at advanced stages (III–IV) in approximately 75% of patients.^1^ The identification of the molecular subsets of NSCLC and the rapid emergence of targeted therapies have stressed molecular pathology laboratories to develop and integrate testing for multiple biomarkers in routine practice, each with inherent analytical challenges. In addition to immunohistochemistry (IHC)-based programmed death-ligand 1 testing, the range of relevant molecular alterations for clinical management is constantly expanding. The alterations with the highest clinical interest are found in several genes (*EGFR*, *ALK*, *ROS1*, *BRAF*, *RET*, *ERBB2*, *MET*, *KRAS*, and *NTRKs*) and consist of various single nucleotide variants (SNVs), insertions and deletions (indels), copy number variations, and oncogenic fusions and isoforms.^2^^,^^3^

Although next-generation sequencing (NGS) is increasingly used to address multigene testing in routine molecular diagnosis, the capacity to capture every type of genomic alteration using a single chemistry remains challenging. NGS assays within reach of hospital-based laboratories are mostly amplicon based and require managing DNA and RNA workflows separately to ensure proper detection of mutations and fusions, respectively. Hybrid-capture panels offer the possibility to deal with a single analyte, but even large comprehensive panels are subject to miss fusions and alternative splicing events, as intron length and repetitive regions are factors complicating the capture of relevant genomic regions.^4^^,^^5^ Although sequential DNA and RNA-NGS are being proposed as the optimal strategy to capture all possible alterations,^6^ this entails a risk for tissue exhaustion inherent to small specimens and increased overall turnaround time, laboratory workload, and costs. All these factors must be considered along with analytical performance when selecting the proper NGS strategy for first-line molecular diagnosis, such as for NSCLC, a tumor type with high volume and a crucial need for robust oncogenic fusion detection.

Currently, the availability and funding of NGS for standard diagnostic evaluation remain poorly described,^7^ and expansion of molecular testing beyond *EGFR*, *ALK*, and *ROS1* for patients with NSCLC persists an unsatisfied challenge in several countries and community settings.^8^^,^^9^ Comprehensive molecular profiling was found to have its impact for discovery of potentially targetable genetic alterations beyond standard of care for metastatic lung adenocarcinoma.^10^ Nevertheless, detection of genomic alterations of uncertain clinical significance challenges public health system evaluation schemes, where in some jurisdictions, diagnostic test approval is tied to decisions regarding reimbursement of therapeutic agents. Mostly owing to the incremental cost of its technology, access to NGS testing remains unequally accessible, often developed on a research backbone, and tumor-focused assays with emphasis on actionable targets are potential strategy to help fill this gap for patient care.

Here, we present the evaluation of the performance characteristics of a custom-designed anchored, multiplexed, polymerase chain reaction (PCR)-based (AMP) RNA-NGS targeted panel allowing simultaneous detection of clinically relevant hotspot mutations and fusions in NSCLC.

## Materials and Methods

### Panel Design

The assay used is the ArcherDx Fusion Plex Lung (ArcherDX, Boulder, CO) with custom additional targets (Supplementary Table 1). It constitutes an ultratargeted RNA-based panel that uses AMP technology^11^ and NGS to detect gene fusions and selected hotspot SNVs and indels. The design encompasses a pool of 230 gene-specific primers 2 (GSP2, see subsequent discussion), covering four control and 15 target genes focusing on those with approved or emergent clinically actionable alterations in lung cancer. The initial design of Archer Fusion Plex Lung was minimally customized by the manufacturer by adding primers to cover *ERBB2* (fusions and hotspot mutations in exons 8 and 20). The assay allows detection of both known and unknown fusion partners and relevant actionable SNV/indel from a single workflow on the basis of RNA sequencing (RNA-seq), using total nucleic acids (TNAs) extracted from formalin-fixed, paraffin-embedded (FFPE) tissue as a starting material.

### Sample Selection and Controls

All samples were from patients who underwent interventional bronchoscopy or surgical resection for NSCLC followed by biomarker testing at the request of referring physician at the Institut Universitaire de Cardiologie et de Pneumologie de Québec—Université Laval (Quebec City, Canada). The study was approved by the Institut Universitaire de Cardiologie et de Pneumologie de Québec—Université Laval ethics committee (#2019-3183-21730). Representative FFPE specimens were selected for this validation set, including cytology cell blocks, small biopsies, and surgical resections. All samples had known alterations characterized previously by another established molecular method. Those consisted of fluorescence in situ hybridization (FISH) with *ALK*, *ROS1*, *RET*, or *NTRK1* probes (SureFISH, Agilent, Mississauga, Canada), *EGFR* RGQ PCR kit ran on a Rotor-Gene Q (Qiagen, Toronto, Canada), or digital droplet PCR *BRAF* V600 or *KRAS* G12/G13 screening kits performed on a QX200 system (Bio-Rad Laboratories, Hercules, CA). A subset of the case was also sequenced using QIAseq-targeted DNA scan technology (Qiagen, Toronto, Canada). Hematoxylin and eosin-stained slides were reviewed for tumor content evaluation by a pathologist before extraction. Decalcified specimens were excluded from this set. Samples selected within the prospective cohort were tested by single-gene assay, including concomitant *EGFR* RGQ and IHC for *ALK* with clone 5A4 (Biocare, Markham, Canada) and *ROS1* with clone D4D6 (CST, Danvers, NH) performed on a Dako Autostainer (Agilent), followed by FISH in equivocal cases.

Reference material included commercial FFPE tumor fusion control (Seraseq FFPE Fusion RNA Mix v4; Seracare, MA) in addition to a collection of cell lines from ATCC (Manassas, VA: NCI-H1975, *ELM4-ALK* Fusion-A549, LC-2/ad, and U118) or Leibniz Institute (Brunswick, Germany: HCC78). Cell lines were used either alone or admixed with each other in formalin-fixed and paraffin-embedded cell blocks prepared similarly to clinical cytology specimens.

### Nucleic Acid Extraction

TNA extraction was performed using two to four FFPE sections of 20 μm with the Qiagen Allprep DNA/RNA FFPE kit (Qiagen, Toronto, Canada). Samples were quantified using the Qubit RNA HS Assay Kit on a Qubit 3.0 fluorometer (Thermo Fisher Scientific, Burlington, Canada). When possible, the maximum input material recommended by the manufacturer was used (250 ng TNA on the basis of RNA quantification), but samples under this threshold were also included to explore this factor on the assay’s performance and sensitivity.

### Library Preparation, Sequencing, and Bioinformatic Analysis

Libraries were prepared using the Archer FusionPlex reagent kit for Illumina (San Diego, CA) according to the manufacturer’s instructions. Briefly, cDNA is synthesized from the RNA using random priming and then undergoes end repair, dA tailing, and adapter ligation with Illumina molecular barcode adapters, which allow for read deduplication and quantitative analysis. Magnetic beads (Macherey-Nagel, Allentown, PA) are used for cleanups after every enzymatic step. The ligated fragments are processed through two rounds of PCR amplification using two sets of GSP and universal primers complementary to the Illumina adapters. The GSP2 pool in PCR2 consists of a nested pool designed 3’ downstream of GSP1. After completion of the two PCR steps, libraries are quantified using NEBNext Library Quant Kit for Illumina (NEB, Ipswich, MA) and run on a Bioanalyzer high-sensitivity DNA chip (Agilent, Mississauga, Canada). They are pooled at equimolar concentration and sequenced on Illumina MiniSeq at 2 × 150 base pair. The FASTQ files generated by the bcl2fastq conversion software (Illumina, San Diego, CA) are then uploaded into the Archer data analysis pipeline (Archer Analysis Software, version 6.2.7).

## Sequencing Quality Controls and Variant Call Criteria

In this assay, the quality of first-strand cDNA synthesis is used as an indicator of RNA quality before library preparation and sequencing. It is evaluated using the Archer PreSeq RNA quality control (QC) Assay (Pre-Seq Ct score), a quantitative PCR-based method. Another key assay metrics is provided on the basis of a pool of GSPs included in the design targeting four control gene transcripts (*CHMP2A*, *GPI*, *RAB7A*, and *VCP*). The Fusion QC score represents the average number of RNA unique start sites calculated per control GSP2. It must be superior to 10 to support enough RNA quality on the assay targets. The Variations QC score represents the average number of DNA or ambiguous unique start sites calculated per GSP2 across the entire panel. Other raw metrics that were monitored included percentage of RNA reads. A combined score integrating preanalytical factors and assay quality metrics on the basis of the observations herein and other unpublished familiarization data was developed to increase the likelihood of assay success: it integrates factors of minimal TNA input (≥100 ng), sample age of FFPE specimen (<4 y old), Pre-Seq Ct score (<25), and Fusion QC score (≥10).

The criteria for calling a positive fusion were five or more unique supporting RNA reads and three or more unique starting sites (SSs) among the reads and an in-frame sequence. The minimal criteria for calling a positive hotspot variant were 5% or more variant allele frequency (VAF), five or more alternate observation, three or more unique start site supporting the variant, and 100 or more read depth; all known hotspot variants were reviewed manually on the Archer Analysis portal and on bam files using Integrative Genomics Viewer (https://software.broadinstitute.org/software/igv/).

### Statistics

Correlation, contingency analysis with Fisher’s exact test, chi-square test, and *t* test were performed using GraphPad Prism, version 9.1.0 (GraphPad Software, San Diego, CA).

## Results

### Sample Characteristics and Overall Performance

**Figure 1:**
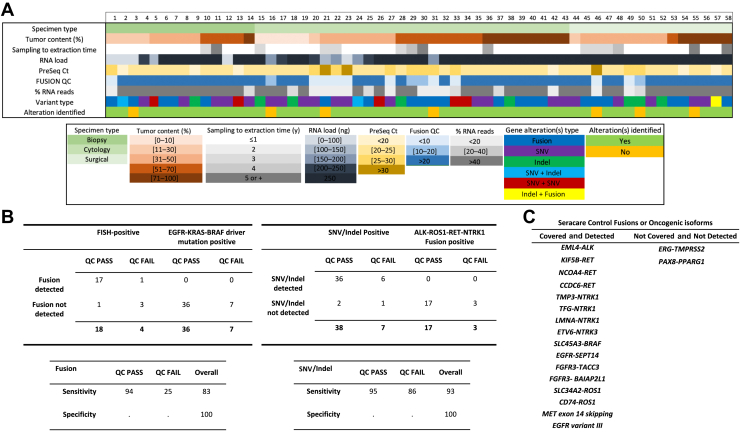
(*A*) Summary of specimen characteristics and assay metrics of the NSCLC samples used for validation of the Archer Fusion Plex Lung assay on FFPE specimens, with each column representing an individual sample. (*B*) The accuracy, on the basis of the target type, is summarized with breakdown of the result of the main QC of the assay, Fusion QC. Positive samples for SNVs/indels were considered true-negative for fusion detection and vice versa. (*C*) Commercial control (Seraseq FFPE Fusion RNA Mix version 4) was used to evaluate the capacity to detect a larger diversity of fusion variants and revealed perfect sensitivity and specificity for the covered gene targets. FFPE, formalin-fixed, paraffin-embedded; FISH, fluorescence in situ hybridization; indel, insertion/deletion; QC, quality control; SNV, single nucleotide variant.

The performance of this customized Archer Fusion Plex Lung panel was evaluated on commercial reference material, characterized cell lines, and 58 individual FFPE NSCLC samples with known SNVs, indels, rearrangements, or splice variant relevant to NSCLC and covered by the panel design (Supplementary Table 2). Most NSCLC samples used in this validation set were small specimens, including most cytology FFPE cell blocks (n = 29; 50%), 1 year old or less (n = 43; 74%), and had tumor content of at least 20% (n = 50; 86%) (Table 1). Most samples met the minimal TNA input (250 ng on the basis of RNA quantification) recommended by the manufacturer’s protocol (n = 44; 74%). The average number of RNA unique start sites calculated per control GSP2, or Fusion QC score, passed the minimal threshold (≥10) in 48 samples (83%). Samples used in this set were processed completely regardless of metrics to evaluate the impact of suboptimal assay parameters in the overall capacity to detect known alterations. Further breakdown of preanalytical, sequencing metrics and results per targeted variant(s) is provided in Figure 1*A*.

**Table 1:** Summary of FFPE Validation Sample Characteristics and Assay Metrics

Characteristics	No. (%)	Fusion QC Pass (%)	*p*^a^	Alteration detected (%)	*p*^a^
Specimen type					
Biopsy	14 (24)	13 (93)	0.35	13 (93)	0.52
Surgical	15 (26)	13 (87)		12 (80)	
Cytology	29 (50)	22 (76)		26 (90)	
Number of variants per sample					
1	49 (84)	40 (82)	>0.99	42 (86)	0.58
2	9 (16)	8 (89)		9 (100)	
Variant type (n = 67)					
SNV/indel	45 (67)	38 (84)	>0.99	42 (93)	0.21
Fusion	22 (33)	20 (82)		20 (82)	
Tumor content (%)					
≤20	8 (14)	7 (88)	>0.99	6 (75)	0.25
≥20	50 (86)	41 (82)		45 (90)	
Sample age (y)					
≤1	43 (74)	36 (84)	0.71	39 (91)	0.36
2–5	15 (26)	12 (80)		12 (80)	
TNA load (ng)					
<250	15 (26)	9 (60)	0.02	11 (73)	0.07
250	43 (74)	39 (91)		40 (93)	
Pre-Seq Ct score					
<25	44 (76)	44 (100)	<0.001	42 (95)	0.007
≥25	14 (24)	4 (29)		9 (64)	
Fusion QC score					
<10 (Fail)	10 (17)	—		6 (60)	0.01
≥10 (Pass)	48 (83)	—		45 (94)	
RNA reads (%)					
<40	18 (31)	8 (44)	<0.001	14 (78)	0.19
≥40	40 (69)	40 (100)		37 (93)	
QC metrics combination: sample age ≤4 y and TNA >100 ng and Pre-seq < 25 and fusion QC >10					
Yes	38 (66)			38 (100)	0.0003
No	20 (34)	—		13 (65)	

FFPE, formalin-fixed, paraffin-embedded; indel: insertion/deletion; QC, quality control; seq, sequencing; SNV, single nucleotide variant; TNA, total nucleic acid.

a Fisher’s exact test or chi-square (specimen type).

As expected, the accuracy of the assay for fusion or SNV/indel detection on archival specimens was affected by the Fusion QC, the assay’s main sequencing metrics (Table 1 and Fig. 1*B*). The sensitivity for fusion detection and SNV/indel reached 94% and 95% when this metrics was met but fell to 25% and 86% when failed, respectively (Fig. 1*B*). Samples with hotspot mutations in *EGFR*, *KRAS*, or *BRAF* were used as negative controls for fusions and vice versa, to capitalize on known mutual exclusivity of key oncogenic drivers in NSCLC; the specificity of the assay was 100% for both types of alterations. In complement, a commercial control containing a mixture of characterized alterations allowed testing the assay’s capacity to detect a wider variety of gene fusion partners, targets, and oncogenic isoforms not already covered with validation samples (Fig. 1*C*).

Concordantly, a Pre-Seq Ct score above 25 was associated with lower proportion of samples revealing success of Fusion QC and ability to detect the targeted alteration (Table 1). Supplementary Figure 1 reveals how Fusion QC correlates with Pre-Seq Ct overall and with the percent of RNA reads, these being general indicators of RNA quality. TNA input below the maximum quantity recommended by the protocol was also associated with a lower Fusion QC pass rate (Table 1). Key preanalytical factors and assay metrics with arbitrary thresholds derived from the observations herein were selected together in a combined score (TNA input higher than 100 ng, samples less than 4 years old, Pre-Seq Ct score lower than 25, and Fusion QC ≥10), then providing a perfect (100%) concordance (Table 1).

Although a high percentage of unique RNA reads on the target region was constantly observed using this assay (>90%, data not found), efficiency of primers specific to target exons for clinically relevant hotspot SNVs/indels and fusions was also evaluated. As expected from the similar accuracy rate inferred from Figure 1*B* for both types of alterations, the distribution of unique reads per GSP2 primers was relatively balanced between exons intended to cover hotspot SNVs/indels, fusions, and control genes (Supplementary Figure 2*A*), being slightly higher when averaged on exons targeting mutations than fusions (t = 3.17; *p* = 0.02). Supplementary Figure 2*B* and *C* reveals the range of read depth and VAF observed for targeted hotspot SNVs and indels. Known SNVs were detected with a mean read depth of 754 (SD = 1217) and mean VAF of 41 (SD = 30), whereas known indels had mean read depth of 1083 (SD = 1984) and mean VAF of 34 (SD = 23), not significantly different (*p* = 0.53 and 0.51 for read depth and VAF, respectively). For a subset of cases where VAF was available from another method (either DNA-based NGS or droplet digital PCR), there was a fair correlation (*R*^2^ = 0.86) with the VAF provided by the FusionPlex Lung assay (Supplementary Fig. 2*D*). The samples with VAF estimates larger than 5% between methods had a significantly lower proportion of DNA reads for supporting the call on the corresponding GSP2 when compared with samples with a less than 5% difference (12% and 55%, respectively) (Supplementary Fig. 2*E*).

### Limit of Detection

To determine the limit of detection of the assay, we used a mixture of known fusion and SNV-characterized cell lines fixed in formalin and processed in paraffin cell blocks as routine cytology specimens in our laboratory. First, libraries were prepared with decremental input of TNA (Table 2). Fusions of QC values all met the threshold but dropped linearly with the decreasing amount of TNA loaded. The two fusions with higher levels of unique SSs were detected with input as low as 20 ng, whereas the two ones with lower levels of SS were not detected below 100 ng input. The SNV/indel coverage also decreased proportionally to the TNA amount. When detected, fusion percent reads and VAF remained in the same range throughout the various inputs used.

**Table 2:** Summary of Total Nucleic Acid Input Variation Experiment

Input (ng)	*SLC34A2-ROS1*		*CCDC6-RET*		*GOPC-ROS1*		*ALK-EML4*		*KRAS* G12S		*EGFR* *L747_P753del*	
	SS	URR (%)	SS	URR (%)	SS	URR (%)	SS	URR (%)	Cov. (AO)	VAF	Cov. (AO)	VAF
250	52	107 (90)	40	120 (97)	13	35 (21)	8	29 (28)	266 (58)	21.8	10,319 (6767)	65.6
150	35	53 (91)	26	53 (93)	9	12 (20)	10	19 (37)	167 (30)	18.0	—	—
100	34	52 (80)	23	43 (88)	9	20 (33)	9	20 (39)	112 (20)	17.9	—	—
75	1	20 (91)	13	24 (96)	—	—	—	—	62 (13)	21.0	3567 (2543)	71.3
20	7	10 (91)	3	6 (86)	—	—	—	—	—	—	1310 (928)	70.8
10	—	—	—	—	—	—	—	—	—	—	—	—
Mean % (SD)	92 (5)		89 (5)		25(8)		34 (6)		20 (2)		69 (3)	

%, % RNA reads; AO, total numbers of reads that support the variant (alternate observation); Cov., coverage; SS, starting site; URR, unique RNA read; VAF, variant allele frequency.

Libraries were also prepared by serial dilutions of samples along with previously tested sample TNA negative for the targeted known alterations (Tables 3 and 4). Numbers of fusion transcripts SSs, reads supporting the variant, and VAF decreased linearly with serial dilutions. As observed for decremental input, detection of fusion transcripts was lost more rapidly when starting with lower number of SS.

**Table 3:** Summary of Total Nucleic Acid Dilution Experiment for Fusion Detection

Fusion	*SLC34A2-ROS1*		*CCDC6-RET*		*GOPC-ROS1*		*ALK-EML4*	
Concentration, %	SS	URR (%)	SS	URR (%)	SS	URR (%)	SS	URR (%)
100	160	971 (91.6)	50	124 (97.6)	21	60 (4.8)	11	21 (14.6)
50	96	266 (91.1)	19	32 (91.4)	9	20 (5.8)	—	—
25	54	115 (94.3)	14	19 (90.5)	3	7 (4.5)	—	—
12.5	39	68 (85.0)	7	12 (70.6)	—	—	—	—

%, % RNA reads; SS, starting site; URR, unique RNA read.

**Table 4:** Summary of Total Nucleic Acid Dilution Experiment for SNV/Indel Detection

Gene Variant	*EGFR* L858R c.2573T>G		*EGFR* T790M c.2369C>T		*KRAS* G12S c.34G>A		*EGFR L747_P753del* c.2240_2257del	
Concentration, %	Cov. (AO)	VAF	Cov. (AO)	VAF	Cov. (AO)	VAF	Cov. (AO)	VAF
100	4369 (961)	22.2	2050 (450)	22.0	523 (104)	19.9	10,319 (6767)	65.6
50	2287 (243)	10.6	997 (113)	11.3	512 (37)	7.2	—	—
25	2339 (114)	4.9	969 (53)	5.5	626 (20)	3.2	1723 (1123)	65.2
12.5	1905 (70)	2.4	1096 (24)	2.2	711 (6)	0.8	1126 (615)	54.6
10	2178 (11)	0.5	365 (2)	0.6	—	—	1189 (600)	50.5
5	2341(4)	0.2	429 (4)	0.9	—	—	833 (303)	36.4
2.5	2222 (5)	0.2	408 (1)	0.3	—	—	763 (176)	23.1
1	1977 (0)	0	422 (2)	0.5	—	—	707 (80)	11.3

AO, total numbers of reads that support the variant (alternate observation); Cov., coverage; indel, insertion/deletion; SNV, single nucleotide variant; VAF, variant allele frequency.

### Reproducibility

To determine the reproducibility of this assay, we analyzed fusion- and SNV-positive samples as triplicate in the same run (intrarun reproducibility) and in three to five different runs (interrun reproducibility). The concordance of both fusion and SNV detection was perfect across the replicates. There was also high reproducibility in the numbers of SS or unique reads for fusion transcripts and for the coverage, VAF, and numbers of reads supporting the variant for SNVs/indels (Supplementary Fig. 3).

### Prospective Clinical Sample Experience

**Figure 2:**
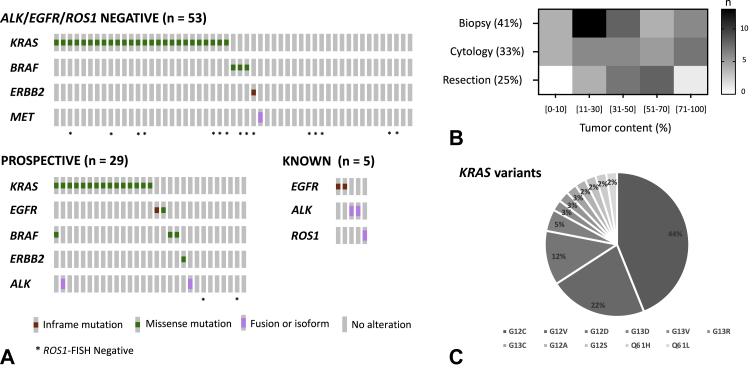
(*A*) Oncoprints revealing the driver alterations identified using Archer Fusion Plex Lung in a prospective set consisting of three different groups of NSCLC samples. (*B*) Heatmap revealing the number of samples according to specimen type and estimated tumor percentage. (*C*) Distribution of *KRAS* variants identified across the entire cohort. FISH, fluorescence in situ hybridization.

The validity and added value of using a highly focused panel for identification of clinically relevant driver alterations were further evaluated on prospective routine NSCLC samples (Fig. 2). Those consisted of three different sets of representative NSCLC clinical sample characteristics, with a predominance of small samples (41% biopsy and 33% cytology) and tumor content covering a wide range (Fig. 2*B*). The first set included 53 samples negative for *EGFR*-activating mutations and *ALK* or *ROS1* fusions, as evaluated by single-gene testing (SGT), enriched in tumors that underwent *ROS1* FISH on the basis of equivocal IHC staining for *ROS1* (15 of 53; 28%). Another set consisted of 29 samples reflex-tested by NGS in parallel to SGT. These were completed with five additional samples with known *EGFR*, *ALK*, or *ROS1* alterations. Driver alterations in key NSCLC genes were found in 31 of 53 (58%) and 23 of 29 (79%) unknown cohorts, respectively (Fig. 2*A*). All *EGFR*, *ALK*, and *ROS1* results obtained with SGT were concordant, with one case of *ALK* fusion revealing a concomitant *KRAS* mutation additionally. As expected from the known prevalence in NSCLC, hotspot driver mutations in *KRAS* were the most often found, where p.G12C represented 44% of *KRAS* variants (Fig. 2*C*). Other potentially actionable variants outside of *EGFR*, *ALK*, and *ROS1* included *BRAF* p.V600E (n = 1/3 *BRAF* mutations), *ERBB2* exon 20 insertion (n = 1), or S310F (n = 1), and *MET* exon 14 skipping alteration (n = 1). Altogether, potentially actionable variants were found in 26 of 82 unknown cases (32%), namely 14 of 29 (48%) when done reflex in parallel to SGT and 12 of 53 (23%) in the cohort of cases negative for *ALK*, *EGFR*, and *ROS1* alterations.

## Discussion

This study reports the analytical performance of a 15-gene customized version of an NGS assay using AMP technology and focused on NSCLC targets. This ultratargeted assay on the basis of RNA is intended for first-line simultaneous detection of clinically actionable oncogenic fusions/isoforms and hotspot SNVs/indels in key genes for NSCLC, with a single workflow for library preparation. The data presented establish the performance of the assay across different types of clinical FFPE specimens, providing that key metrics regarding RNA quality are monitored. More specifically, the maximum recommended TNA input (250 ng) should be used when possible (not less than 100 ng). In addition, use of complementary testing is warranted when interpreting a negative result if RNA quality, on the basis of the PreSeq Ct score and the sequencing Fusion QC assay, is under optimal threshold values.

The data obtained during the course of this technical validation reveal some characteristics relevant to RNA-based NGS assays. First, RNA quality and quantity are crucial to obtain success as reflected by the importance of the Fusion QC metrics in the Archer’s design. Although not absolutely precluding detection of fusions or mutations, failure to meet this metrics is associated with a much lower probability to detect an alteration when present. Consequently, samples negative for alterations in the context of suboptimal metrics or coverage should be considered uninformative and undergo additional testing with complementary method, similarly to a DNA-based NGS assay. Interestingly, the use of TNA as starting input provides an additional indirect appreciation of RNA quality, as large differences in the number and percent of reads obtained from RNA and DNA can be identified in a sample with low RNA quality for each target.

Second, the validation of fusion detection with RNA-NGS assays is challenging owing to variable expression levels found in tumor samples. Large differences in the level of expression of fusion transcripts affect the ability to determine the limit of detection as linearly as SNV/indels in DNA mutation testing. This factor can attenuate the impact of tumor content and concentration, limiting the ability to evaluate some validation parameters. The dilution experiments found here highlight the importance of using fusion samples with variable starting expression levels in the validation process of such assays. Similarly, the detection of fusions despite failure of the main RNA quality parameter (Fusion QC) suggests an impact of elevated expression.

Third, despite the good correlation observed on a subset of samples matched with DNA-based estimate of VAF for SNV/indels, some samples exhibited larger differences. Factors affecting gene expression, per example owing to *EGFR* amplification, could presumably affect the VAF observed on an RNA-based assay. Nevertheless, this cannot be clearly highlighted as the assay is not intended to use the subset of DNA reads captured to provide amplification data. Furthermore, however, as RNA, DNA, and ambiguous reads captured for every GSP2 are used to call a variant, we noted that the RNA-based estimated of VAF seemed to be closer to the DNA-based one when the proportion of DNA reads, or DNA/RNA reads ratio, was higher.

The performance of AMP-PCR chemistry in solid tumors has been abundantly described for fusion detection^5^^,^^12^^,^^13^ and combined variant/fusion detection using parallel DNA and RNA workflows.^14^^,^^15^ In contrast, the detection of clinically relevant hotspot mutations using RNA-based NGS is very limited and no study was identified for NSCLC with this strategy. Only another observational study was found using RNA-SNV calling on diffuse large B-cell lymphoma, with a panel interrogating hotspot mutations in 35 genes and reporting mutations in 58.5% of cases.^16^

Thus, some factors must be reminded considering the good concordance observed for SNV/indel detection using the current assay. First, although the design covers several exons, the assay is intended to detect only selected hotspot oncogenic driver or resistance mutations. The capacity to detect unique mutations outside of the selected genomic regions interrogated has not been evaluated and is presumably lower than a DNA-based assay given the expected variation on sequencing depth and coverage across the genome for RNA-seq data. This was observed here indirectly with the large spread of read depth on target exons, although the use of molecular indexes in the design likely compensates this factor, abrogating PCR artifacts and allowing detection of SNV/indels at lower coverage (<500 reads). Although similar GSP2 primer coverage was observed across the targeted exons for fusions versus SNVs/indel, suggesting good primer performance overall, only restricted gene regions limited to driver mutations are analyzed. On the samples failing the Fusion QC but correctly calling SNV/indels (n = 6), the proportion of DNA reads was high (72% DNA reads in contrast to 13% in RNA reads on average) regardless of read depth, suggesting DNA reads can partly secure such call when RNA quality is poor. On samples where the SNV/indel were missed (n = 3), one had lack of coverage of the hotspot region (<20 reads), one had no RNA or DNA reads (only ambiguous reads), and another was older than 5 years old (data not shown). Apart from these observations, data from single-cell RNA-seq support that oncogenic or actionable SNVs/indels are detectable in RNA-seq from lung adenocarcinoma, notably, but the selection of proper bioinformatics alignment tools for indel detection is essential.^17^^,^^18^ Our observations here suggest that the Vision variant caller and Archer’s bioinformatics pipeline overall are sufficiently robust for detecting such type of alterations in a clinical setting.

The NGS panel used here offers several advantages regarding its implementation in a clinical laboratory over its ability to replace multiple sequential or parallel PCR-based or in situ assays on the same material. The limited design focuses on the detection of clinically actionable set of gene alterations in NSCLC within a million reads per sample. When combined with programmed death-ligand 1 IHC, it allows to cover all oncogenes either with Food and Drug Administration–approved therapy or recommended by the National Comprehensive Cancer Network guidelines,^19^ going beyond the last Canadian recommendations on biomarker testing for NSCLC.^20^ On the analytical side, it offers the added resolution of partner agnostic detection of fusion and oncogenic isoforms. This aspect gains in interest as characterization of fusion variants to predict clinical response and concerns for missing *MET* exon 14 skipping mutations when using DNA-based NGS only is emerging.^14^^,^^21^ In addition, the focused hotpot approach for SNVs/indel calling reduces the burden of interpreting unknown variants. Although counterintuitive for the NGS technology, it responds to recommendations observed in some jurisdiction to filter out variants not relevant to the clinical indication or tumor type, as reflected in some decisions rendered from health technology assessment bodies.^22^

Among other advantages of this assay, factors simplifying laboratory workflow are of consideration. These include the manufacturer’s user-friendly design and reagent configuration (lyophilized reagents, prepackaged in colored wells of strip tubes) and simplifying handling and training of laboratory technologists for a high-complexity wet-bench procedure. In addition, avoiding the simultaneous or sequential preparation of libraries from DNA and RNA increases overall laboratory handling efficiency and favors delivery of results within a shorter turnaround time. Although NGS reimbursement is not warranted in several jurisdictions, detection of the drug-related relevant hotspot driver mutations and fusions in a single workflow offers an interesting compromise to amalgam appropriate genomic testing within restrictions present in public health systems. Nevertheless, this strategy does not resolve the complexity generated by the companion diagnostic landscape and how some authorities will cope with results coming from assays outside of selected companion assays.

Some of the limitations of this assay, outside its low number of targets, are intrinsically related to the use of RNA as the main analyte. Although not evident from the current limited set including old retrospective samples with rare alterations, RNA is a substrate more labile and sensitive to preanalytical factors than DNA in clinical samples.^23^ RNA degradation increases with the number of years of fixation, and low input amount entails the risk of amplifying this factor.^24^ Although the sequencing success and proportion of driver alteration found in the prospective set herein was high, using representative clinical samples and important proportion of small specimens, small size, and sampling bias limit the capacity to extrapolate the conclusions for large-scale screening. Because of these characteristics, it is necessary to consider integration of complementary conventional testing, both on-slide and DNA-based methods, to rescue minimal gene target assessment (i.e., *EGFR*, *ALK*, and *ROS1*) in samples not meeting the assay metrics. Another unaddressed question by this validation set is the potential of this assay to detect gene amplification, which would need further evaluation of expression data generated within the assay. Despite growing interest, *MET* and *EGFR* amplification detection in NSCLC is mostly found in acquired resistance setting^25^^,^^26^ and cutoff criteria are still not well defined.^27^

Overall technical considerations for which most considerations were directed in this study, the small prospective cohort offers an interesting perspective on the clinical use of this panel. The high percentage of driver alterations identified in both reflex-tested and *EGFR/ALK/ROS1*-negative cases is not surprising owing to the design focused on highly relevant targets in NSCLC. A rate of driver mutations at approximately 60% has been identified when using DNA/RNA-NGS panel in NSCLC,^6^ a percentage not so far from the «reflex» cohort herein (48%) with a much smaller panel. The rate of alterations leading to clinical action, when using current approved drugs, should be similar but requires a larger cohort for characterization using the focused panel presented here.

In conclusion, the data presented in this study describe the performance characteristics of an ultrafocused AMP NGS assay allowing simultaneous detection of SNVs, indels, fusions, and oncogenic isoforms from a single unified RNA-based workflow. It provides important insight in the feasibility to detect hotspot mutations from RNA in a clinical setting. The prospective set of tumors found here does not provide a true incidence of driver alterations in our population owing to the sampling bias. Nevertheless, it provides insight on the high success rate and potential for added value in identification of driver and actionable alterations in NSCLC, even when using a small panel focused on genes with high clinical relevance. The assay used has the potential to replace multiple tests in a unified, single workflow and to provide genomic profiling in a timely manner for patients with NSCLC, especially in administrative settings where molecular testing relevance is tied on therapeutic drug approval scheme.

## CRediT Authorship Contribution Statement

**Patrice Desmeules:** Conceptualization, Funding acquisition, Investigation, Writing - original draft, review, and editing.

**Dominique K. Boudreau:** Data curation, Formal analysis, Methodology, Writing - review and editing.

**Nathalie Bastien:** Data curation, Methodology, Resources, Writing - review and editing.

**Marie-Chloé Boulanger:** Data curation, Formal analysis, Resources, Project administration.

**Yohan Bossé, Philippe Joubert, Christian Couture:** Writing - review and editing.
