# Supplementary material for: Performance of an RNA-Based Next-Generation Sequencing Assay for Combined Detection of Clinically Actionable Fusions and Hotspot Mutations in NSCLC
Source: JTO Clin Res Rep. 2022 Jan 10;3(2):100276. doi: 10.1016/j.jtocrr.2022.100276 (PMC8888203; doi:10.1016/j.jtocrr.2022.100276)
Supplement: Supplementary Tables 1 and 2 and Supplementary Figures 1-3 [file mmc1.pdf]

Supplementary Table 1: Summary of Gene and Exon Content of the of Panel Targets				
**Gene**	RefSeq ID	Target Exons	SNVs/Indels	Fusion/Isoforms
**ALK**	NM_004304	2, 4, 6, 10, 16-23, 26		*
		22, 23, 25	*	
**BRAF**	NM_004333	1-5, 7-16		*
		15	*	
**EGFR**	NM_005228	1, 7-9, 16-21, 24, 25		*
		18-21	*	
**ERBB2**	NM_004448	4, 5, 20, 23-26		*
		8, 20	*	
**FGFR1**	NM_015850	2-12, 17		*
		4	*	
**FGFR2**	NM_000141	2, 3, 6-10, 16, 17		*
		7, 9	*	
**FGFR3**	NM_000142	3, 5-10, 12-18		*
		7, 9, 13, 14, 16	*	
**KRAS**	NM_004985	2-4	*	
**MET**	NM_000245	2, 4-6, 13-17, 21		*
**NRG1**	NM_013962	1-6, 8, 10		*
**NTRK1**	NM_002529	2 ,4 ,6, 8, 10-14		*
		13-14	*	
**NTRK2**	NM_006180	5, 7, 9, 11-18		*
		16-18	*	
**NTRK3**	NM_002530	4, 7, 10, 12-17		*
		15-16	*	
**RET**	NM_020630	2, 4, 6, 8-14		*
		8-16	*	
**ROS1**	NM_002944	2, 4, 7, 31-38		*
		36-38	*	

SNV: single nucleotide variation; Indels: insertion / deletion

Supplementary Table 2: Summary of Reference FFPE Samples used for Validation				
**Alteration type**	Target Genes	Source / Alternative Methods	Fusion QC Pass	Fusion QC Fail
**Single Nucleotide Variation**	EGFR	Qiagen EGFR RGQ PCR (Qiagen DNA scan) NCI-H1975 cell line	16 (4)	3
	BRAF	Bio-Rad V600 ddPCR	7	1
	KRAS	Bio-Rad G12/G13 ddPCR	4	2
		A549 cell line	1	0
**Insertions Deletions**	EGFR	Qiagen EGFR RGQ PCR (Qiagen DNA scan)	10 (5)	1
**Rearrangements**	ALK	FISH (Agilent probes)	11	3
		ELM4-ALK Fusion-A549 cell line	1	0
	ROS1	FISH (Agilent probes)	7	1
		U118 cell line	1	0
		HCC78 cell line	1	0
	RET	FISH (Agilent probes)	2	0
		LC-2/ad cell line	1	0
	NTRK1	FISH (Agilent probes)	1	0
**Oncogenic Fusions and Isoforms**	ALK, RET, ROS1, NTRK1, NTRK2, NTRK3, METex14 FGFR3, BRAF	Seraseq® Fusion RNA Mix v4	1	0

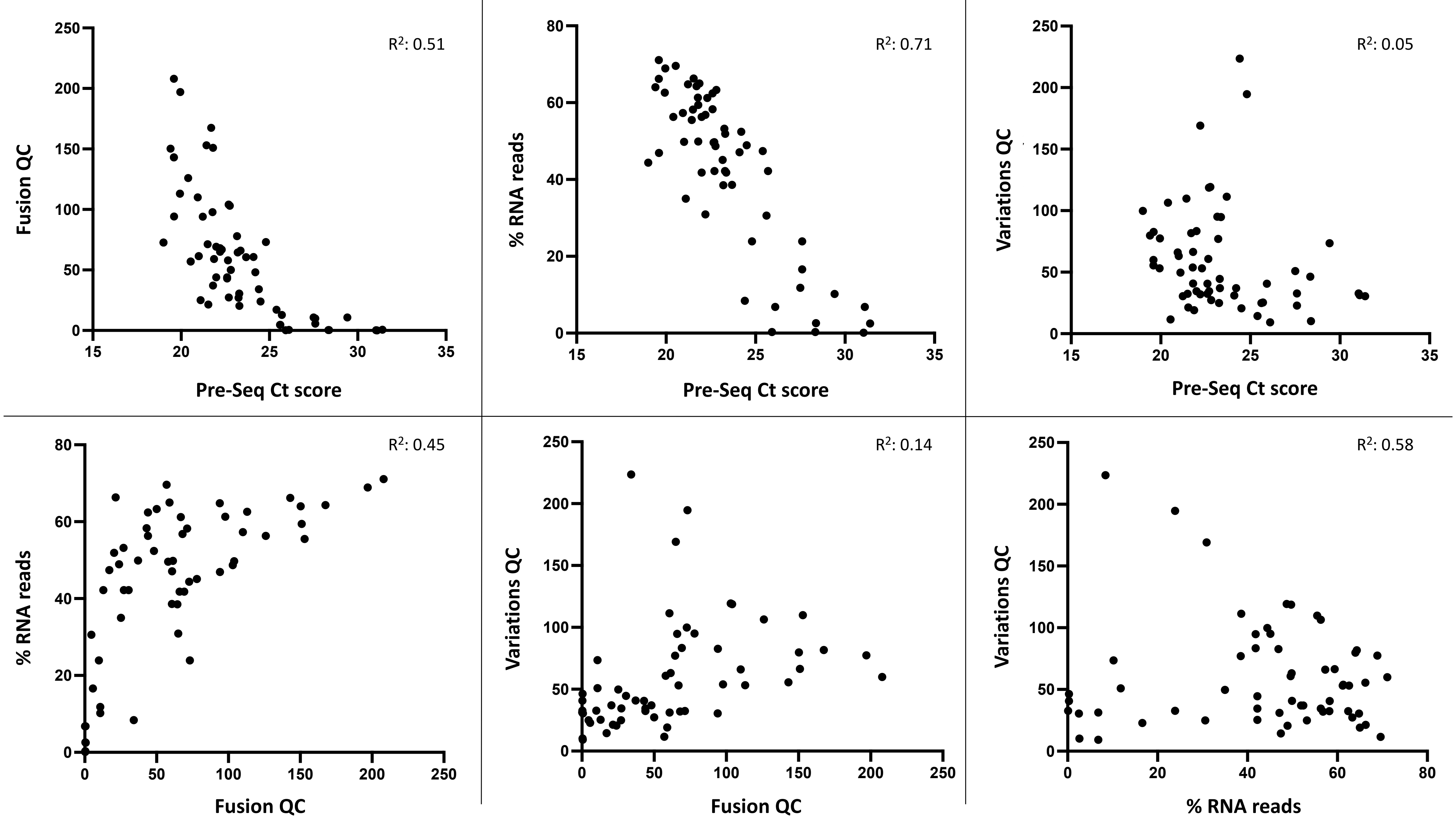

Supplementary Figure 1: Correlation of main Archer Fusion Plex Lung quality control metrics. As expected, high Pre-Seq Ct values inversely correlate with average number of RNA unique start sites per control GSP2 (Fusion QC), while percent RNA reads correlates with both, as they are general indicators of good RNA quality. The average number of DNA or ambiguous unique start sites calculated per GSP2 across the entire panel (Variations QC) similarly correlates with the percent RNA reads.

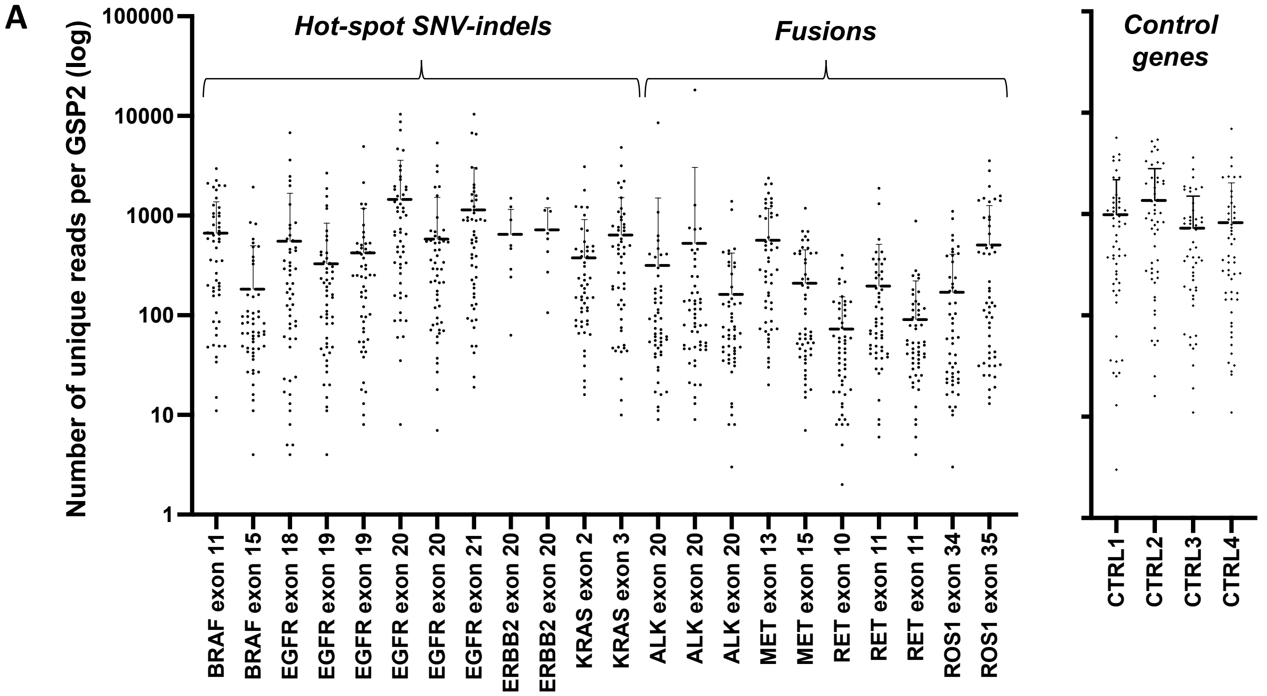

Supplementary Figure 2: Range of: A) unique deduplicated reads per gene specific primer (GSP2) covering the exons with clinically relevant hotspot SNVs/indels or included in canonical fusions of target genes, compared to control genes (plotted in log-units on the Y-axis); B) variant allele frequency (VAF) and C) read depth of known variants detected in six relevant exons with actionable hotspot mutations. D) Correlation between VAF obtained using Archer Fusion Plex Lung (AFPL) and other method, either DNA-based NGS using Qiagen DNA scan or ddPCR, on a subset of cases (n = 21). E) Percentage of reads from DNA and RNA for cases from D according to the difference in VAF obtained between AFPL variant calling or DNA-based calling. Red dots represent indel while black dots represent SNVs. SNV: single nucleotide variation; Indel: insertion or deletion. Bars represent mean and standard deviation.

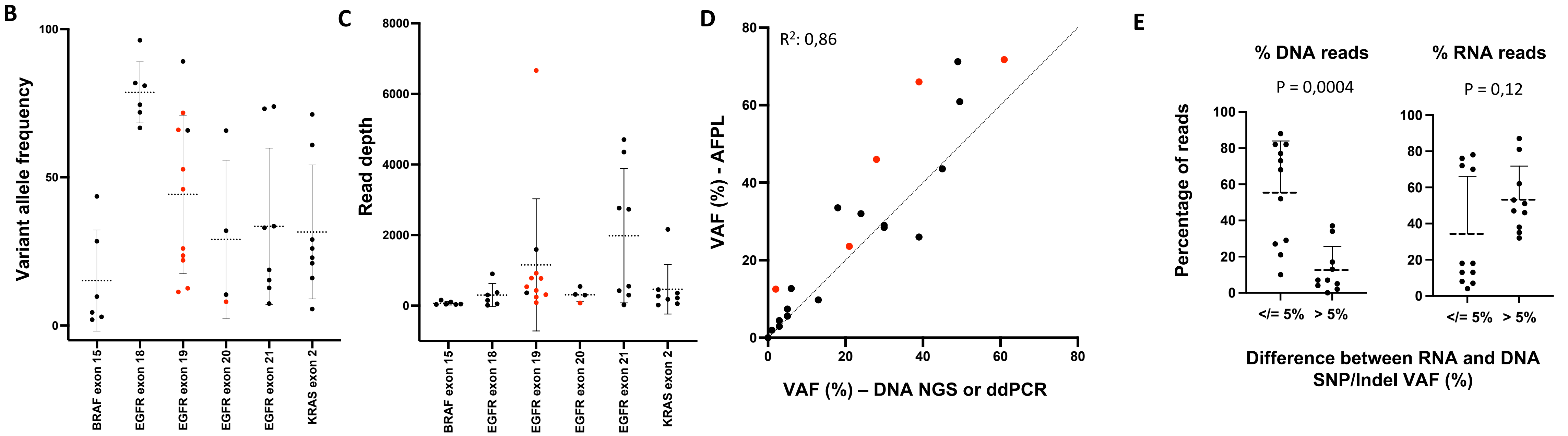

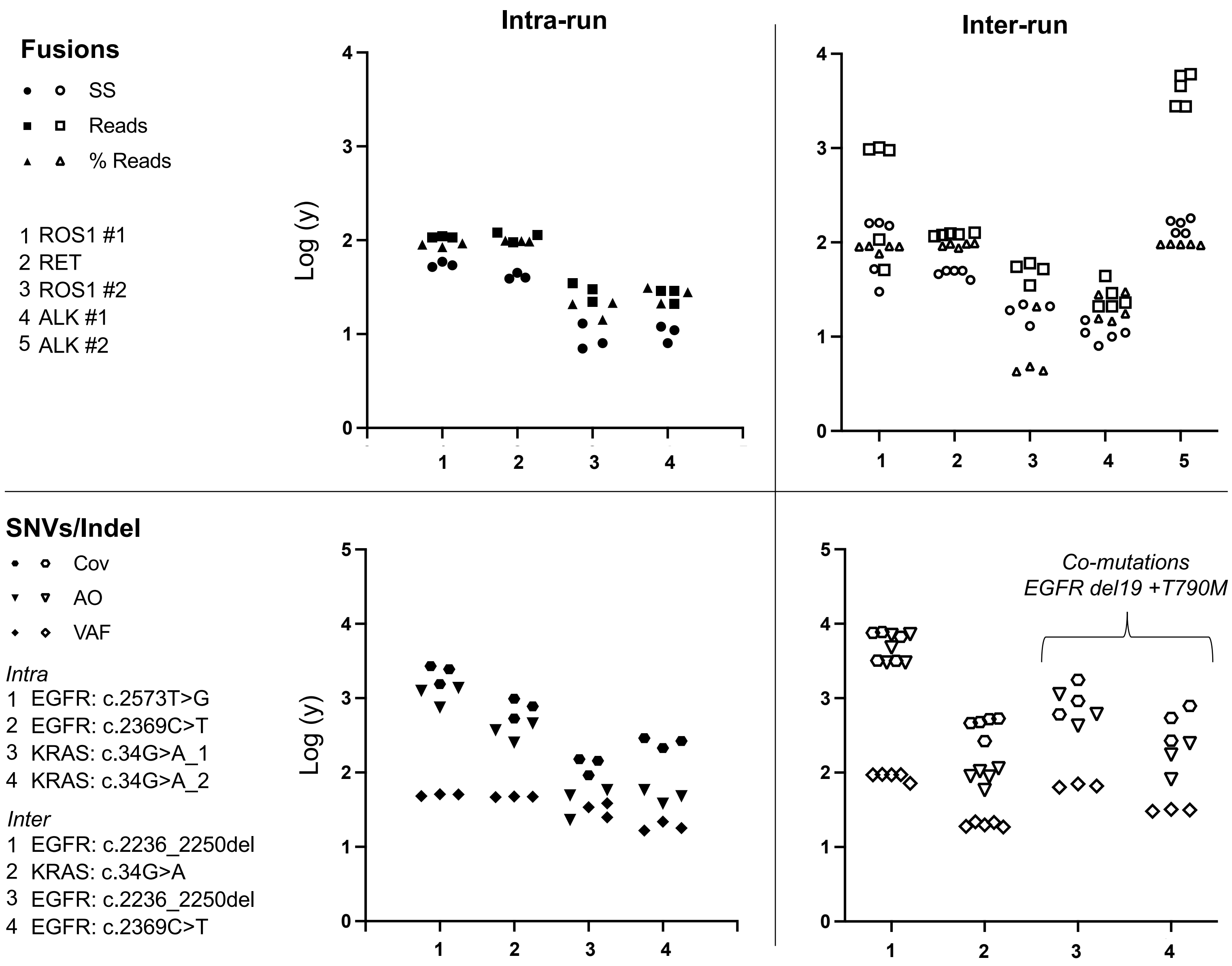

Supplementary Figure 3: Reproducibility of fusion and SNV/indel variant calls comparing inter- and intra-run replicates. The parameters were plotted in log-units on the Y axis: SS: Unique starting site; Reads: unique supporting RNA reads; SNV: single nucleotide variation; Indel: insertion or deletion; Cov: Coverage; AO: Total numbers of reads that support the variant (alternate observation); VAF: Variant allele frequency.
